# Cloning and Functional Analysis of the *SiMAPKKK17* Gene in Foxtail Millet (*Setaria italica*)

**DOI:** 10.3390/plants15071055

**Published:** 2026-03-30

**Authors:** Xinwei Xue, Ankang Mu, Fan Yang, Jialin Zhang, Shi Zhang, Dan Liu, Lei He, Liyan Zhang, Yushan Zhao, Yongping Zhang, Xianrui Wang

**Affiliations:** 1 College of Agronomy, Iner Mongolia Agricultural University, Hohhot 010018, China; xuexinwei@163.com; 2 Chifeng Academy of Agricultural and Animal Husbandry Sciences, Chifeng 024031, China; muankang0104@163.com (A.M.); yf201613@163.com (F.Y.); 18804894322@163.com (J.Z.); zhangshi5256@163.com (S.Z.); liudy2804@163.com (D.L.); helei3379@163.com (L.H.); 15149079150@163.com (L.Z.); 15326761718@163.com (Y.Z.)

**Keywords:** foxtail millet, *SiMAPKKK17*, gene cloning, drought stress, functional analysis

## Abstract

Mitogen-activated protein kinase kinase kinases (MAPKKKs) play important roles in plant responses to abiotic stresses; however, the function of *SiMAPKKK17* in mediating drought tolerance in foxtail millet remains unclear. In this study, the expression pattern, subcellular localization, and biological function of *SiMAPKKK17* were investigated to clarify its role in the drought stress response. Tissue expression analysis showed that *SiMAPKKK17* was expressed across developmental stages and in multiple organs, with the highest transcript level observed at the booting stage and comparatively higher expression in vegetative tissues, including roots, stems, and leaves. Subcellular localization analysis demonstrated that *SiMAPKKK17* was localized to both the plasma membrane and the nucleus, suggesting potential involvement in membrane-associated signal transduction and nuclear regulatory processes. To evaluate its function, foxtail millet lines overexpressing *SiMAPKKK17* were generated and subjected to drought stress. Compared with wild-type plants, the overexpression lines exhibited enhanced drought tolerance, as indicated by greener and more upright upper leaves, higher aboveground fresh weight, greater plant height, and larger leaf area under drought conditions. Transcriptome analysis of OE4 and WT plants under drought stress identified 3919 upregulated genes and 2965 downregulated genes in OE4 compared with WT. These differentially expressed genes were mainly enriched in chloroplast-related cellular components, as well as biological processes and metabolic pathways related to cellular amide metabolism, ion transport, carbon metabolism, photosynthesis, carbon fixation, purine metabolism, and amino acid biosynthesis. Taken together, these results indicate that *SiMAPKKK17* acts as a positive regulator of drought tolerance in foxtail millet, potentially through modulation of photosynthesis- and metabolism-related pathways. This study provides evidence for the molecular mechanisms underlying drought tolerance in foxtail millet and identifies *SiMAPKKK17* as a promising candidate gene for the development of drought-resistant cultivars.

## 1. Introduction

Drought is one of the major abiotic stresses affecting crop production, characterized by widespread occurrence and prolonged duration [1,2,3]. It significantly limits plant growth, development, and yield. According to statistics, drought has caused over 40% of global crop yield reductions or complete crop losses, and the range and severity of its impact continue to intensify, making it one of the most urgent environmental challenges affecting global food security [4,5].

Foxtail millet, a traditional cereal crop originating in China, exhibits high water-use efficiency, strong drought tolerance, and a rich and balanced nutritional composition. It is widely cultivated in arid and semi-arid regions characterized by insufficient heat accumulation and limited water resources and is regarded as a key crop for improving dietary diversity and supporting sustainable dryland agriculture [6,7,8]. Moreover, foxtail millet is a C4 species with a homologous diploid genome, a short growth period, and a relatively small genome, as well as abundant drought-resistance-related genetic resources, making it an important model species for research within the Poaceae family [9,10,11].

In recent years, gene cloning and functional studies in foxtail millet have developed rapidly, leading to the identification of multiple drought-related genes. For example, overexpression of *SiARDP* [12], *SiATG8a* [13], and *SiWLIM2b* [14] has been shown to improve the drought tolerance of foxtail millet. SiNAC18 enhances seed germination and antioxidant capacity under drought conditions by regulating the expression of key genes involved in the abscisic acid (ABA) signaling pathway and oxidative stress responses [15]. Similarly, *SiMYB56* enhances drought tolerance in foxtail millet by regulating lignin biosynthesis and the abscisic acid (ABA) signaling pathway [16]. These findings indicate that the identification and functional characterization of stress-responsive regulatory genes remains an effective strategy for improving drought tolerance in foxtail millet.

Mitogen-activated protein kinases (MAPKs) constitute a conserved “MAPKKK → MAPKK → MAPK” three-tier signaling cascade that converts extracellular signals into intracellular responses through sequential phosphorylation, thereby amplifying and transmitting stress signals. MAPKs function as central signaling hubs in crop responses to abiotic stresses such as drought [17,18,19]. Functional studies of MAPK family members have demonstrated their importance in drought tolerance across crops. In rice, overexpression of *OsMAPK5* increased drought survival by approximately 40% [20]. In wheat, *TaMAPK3* was upregulated under drought stress, and transgenic plants exhibited a 30% increase in proline content and a 25% increase in superoxide dismutase (SOD) activity, thereby significantly enhancing drought tolerance [21]. In maize, overexpression of *ZmMAPK7* reduced chlorophyll degradation under drought, decreasing yield loss by 15–20% [22]. In cotton, *GhMPK7* enhances drought tolerance by promoting the phosphorylation-dependent degradation of the abscisic acid (ABA) signaling negative regulator *GhSDIRIP1* [23].

As critical upstream components of the MAPK cascade, MAPKKKs directly influence signal initiation and specificity. MAPKKKs perceive extracellular cues and activate downstream MAPKKs through mechanisms such as interactions with upstream regulators, autophosphorylation, and conformational changes. They subsequently phosphorylate conserved sites on MAPKKs to trigger the three-tiered kinase cascade. Under drought stress, MAPKKKs participate in drought signaling through the abscisic acid (ABA)-regulated *MAP3K17/18–MKK3–MPK1/2/7/14* cascade in *Arabidopsis*, while the *MAP3K18–MKK3* module positively regulates drought tolerance through ABA-mediated stomatal closure [24]. In rice, overexpression of *OsDSM1*, a MAPKKK protein, enhances the activities of the peroxidases *POX22.3* and *POX8.1*, thereby maintaining reactive oxygen species (ROS) homeostasis and positively regulating drought tolerance [25]. In cotton, the *GhMAP3K15–GhMKK4–GhMPK6* signaling cascade participates in drought-response signaling pathways and enhances drought tolerance by phosphorylating and activating the downstream *GhWRKY59–GhDREB2* regulatory module via an abscisic acid (ABA)-independent pathway [26].

There have been no reports on the role of the foxtail millet MAPKKK gene *SiMAPKKK17* in drought tolerance, and its regulatory mechanisms remain unclear. Based on previous transcriptome analyses of foxtail millet germplasm with different levels of drought tolerance, *SiMAPKKK17*, a mitogen-activated protein kinase kinase kinase (MAPKKK) gene, was identified as a key candidate involved in the drought stress response. In this study, we cloned the *SiMAPKKK17* gene encoding a protein kinase; analyzed its protein sequence, structural characteristics, and evolutionary relationships; and generated foxtail millet plants overexpressing *SiMAPKKK17* via *Agrobacterium-mediated* transformation. The role of *SiMAPKKK17* in the drought stress response was further elucidated through analysis of transgenic plants subjected to drought conditions, thereby providing a theoretical basis for the improvement and development of drought-resistant foxtail millet varieties.

## 2. Results

### 2.1. Cloning and Sequence Analysis of the SiMAPKKK17 Gene

**Figure 1:**
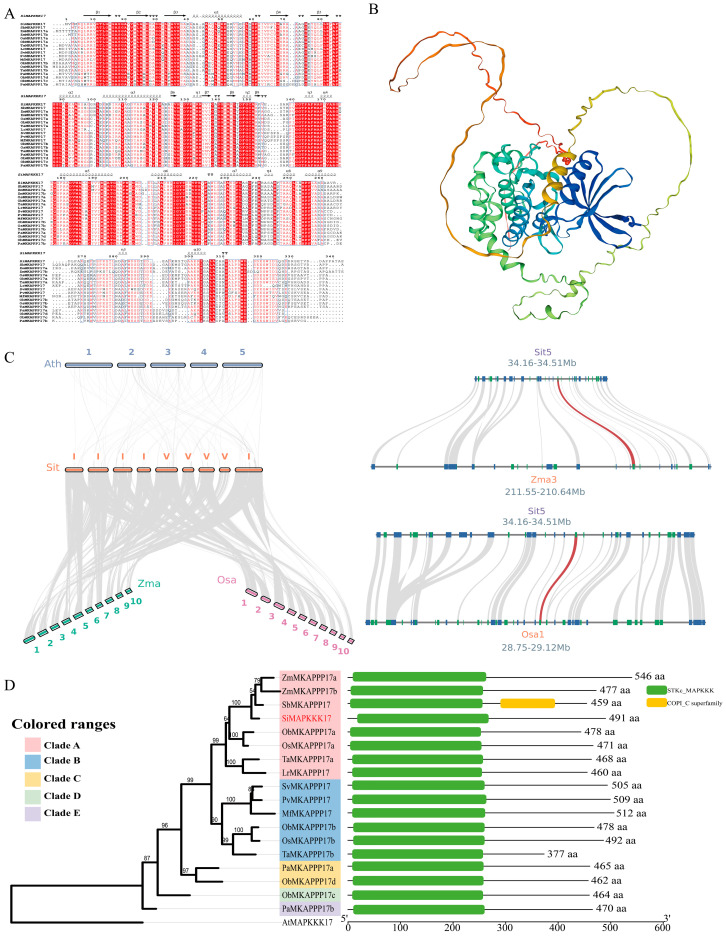
Sequence, structural, syntenic, and phylogenetic analyses of *SiMAPKKK17*. (**A**) Multiple sequence alignment and conserved motif analysis of *SiMAPKKK17* and homologous proteins from representative Poaceae species. Sequences were aligned using MAFFT and visualized using ESPript 3. (**B**) Predicted three-dimensional structure of the *SiMAPKKK17* protein generated by SWISS-MODEL. (**C**) Synteny analysis of *SiMAPKKK17* and related genomic regions. The left panel shows an overview of collinear relationships between the foxtail millet genome and the genomes of maize, rice, and *Arabidopsis*. The right panels show local synteny analysis of the genomic region containing *SiMAPKKK17* in foxtail millet with the corresponding syntenic regions in maize and rice. Red lines indicate the collinear gene pair corresponding to *SiMAPKKK17*, and gray lines indicate other homologous gene pairs in the surrounding regions. (**D**) Phylogenetic relationships and conserved domain organization of *SiMAPKKK17* and related homologs. The phylogenetic tree was constructed using IQ-TREE 3 and visualized using iTOL. Conserved domains were identified using NCBI-CDD and visualized using TBtools(v2.400). Numbers at the nodes indicate branch support values.

Sequence analysis revealed that *SiMAPKKK17* is a typical MAPKKK protein with a highly conserved kinase domain compared with its homologs from representative Poaceae species (Figure 1A). Multiple sequence alignment showed that the ATP-binding site and catalytic motifs were highly conserved among these proteins, indicating strong evolutionary conservation of kinase activity (Figure 1A). Three-dimensional structure prediction further suggested that *SiMAPKKK17* adopts the canonical bilobal structure characteristic of protein kinases, with a compact kinase core and a relatively extended variable region (Figure 1B). To further examine its evolutionary conservation, synteny analysis was performed at both the genome-wide and local genomic locus levels. The overview comparison revealed extensive collinearity between foxtail millet and other representative plant species, including maize, rice, and *Arabidopsis* (Figure 1C, left). More importantly, local synteny analysis focusing on the genomic region surrounding *SiMAPKKK17* revealed conserved collinear relationships between the *SiMAPKKK17* locus on foxtail millet chromosome 5 and the corresponding syntenic regions on maize chromosome 3 and rice chromosome 1 (Figure 1C, right), indicating that the genomic context of *SiMAPKKK17* is conserved among species of the Poaceae family. Conserved domain analysis showed that *SiMAPKKK17* contains a typical STKc_MAPKKK kinase domain and a coiled-coil region, consistent with the structural characteristics of MAPKKK family proteins (Figure 1D). Phylogenetic analysis demonstrated that *SiMAPKKK17* clusters with homologous proteins from closely related Poaceae species, particularly maize and sorghum, with strong bootstrap support, indicating that *SiMAPKKK17* is evolutionarily conserved within the Poaceae family (Figure 1D).

### 2.2. Expression of SiMAPKKK17 Gene in Different Organs of Foxtail Millet

**Figure 2:**
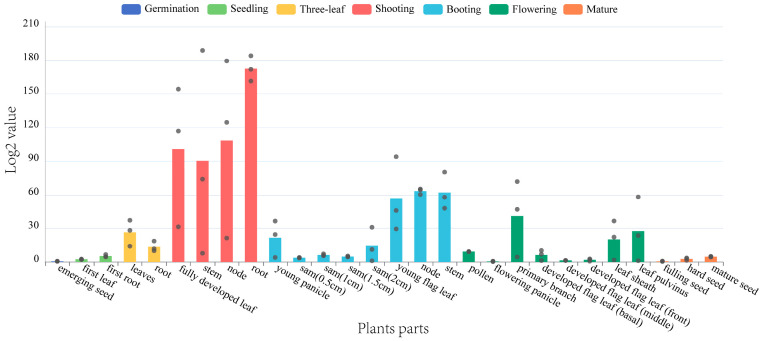
Tissue-specific expression patterns of *SiMAPKKK17* in foxtail millet at different developmental stages and in different organs, determined by qRT-PCR. Relative expression levels were normalized to the internal reference gene and are shown as mean.

Tissue expression analysis revealed that *SiMAPKKK17* exhibits distinct spatiotemporal expression patterns in foxtail millet, with detectable expression across multiple developmental stages and organs. As shown in Figure 2, transcript levels were lowest during the germination and seedling stages, peaked at the booting stage, and subsequently declined during the flowering and maturation stages. Among different organs, *SiMAPKKK17* transcript levels were significantly higher in vegetative tissues (roots, stems, and leaves) than in reproductive organs.

### 2.3. Subcellular Localization of SiMAPKKK17 Protein

**Figure 3:**
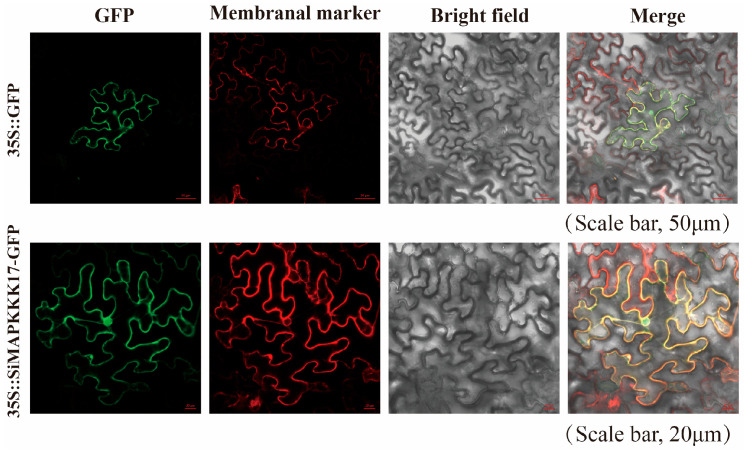
Subcellular localization of *SiMAPKKK17* in *Nicotiana benthamiana* leaves. The pCAMBIA2300-GFP*-SiMAPKKK17* fusion construct and the empty pCAMBIA2300-GFP vector were transiently expressed in *N. benthamiana* leaves by *Agrobacterium-mediated* infiltration. Fluorescence signals were observed 36–48 h after infiltration using confocal laser scanning microscopy (LSM 980, Carl Zeiss, Jena, Germany). GFP and mCherry signals were detected using the 488 nm and 561 nm channels, respectively. PIP2A-mCherry was used as the plasma membrane marker. For plasmolysis analysis, leaf tissues were treated with 1 M mannitol for approximately 10 min before imaging. Compared with the empty vector control, the *SiMAPKKK17*-GFP signal was enriched at the cell periphery and also detected in the nucleus, indicating localization in the plasma membrane-associated region and nucleus.

The subcellular localization of *SiMAPKKK17* in foxtail millet was initially predicted using the Plant-mPLoc web tool, which suggested that *SiMAPKKK17* is predominantly localized in the nucleus. To validate this prediction, a pCAMBIA2300-GFP-*SiMAPKKK17* fusion construct was generated and transiently expressed in *N. benthamiana* leaves, with the empty pCAMBIA2300-GFP vector serving as the control. As shown in Figure 3, the GFP signal from the empty vector was distributed throughout the cell, whereas the fluorescence signal of SiMAPKKK17-GFP was enriched at the cell periphery and also detectable in the nucleus. Combined with plasmolysis assays, these results indicate that *SiMAPKKK17* is localized to both the plasma membrane and the nucleus, suggesting its potential involvement in membrane-associated signal transduction and nuclear regulatory processes.

### 2.4. Verification and Characterization of SiMAPKKK17 Overexpression Transgenic Lines

**Figure 4:**
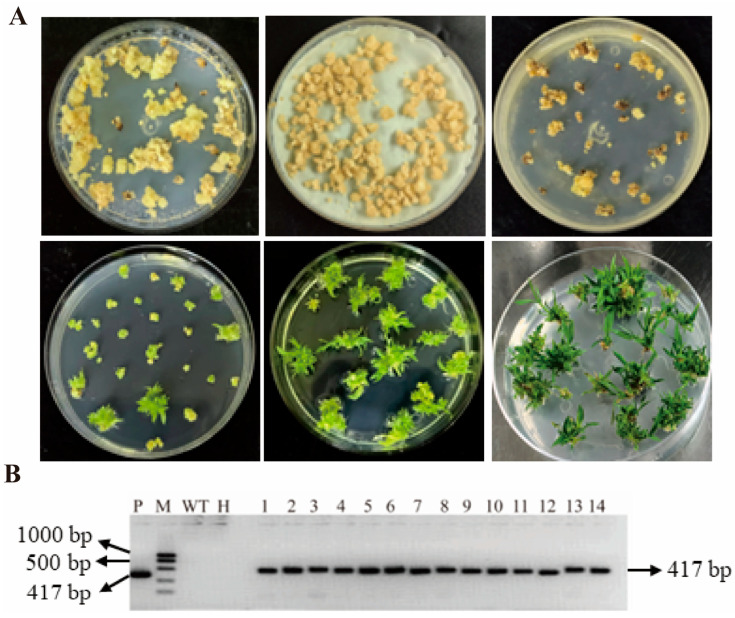
Construction of the *SiMAPKKK17* Overexpression Vector and Genetic Transformation. (**A**) Transformation process of *SiMAPKKK17* expression in millet. (**B**) PCR identification of transgenic foxtail millet plants using hygromycin resistance gene-specific primers. A specific 417 bp fragment was detected in positive transgenic plants and the plasmid positive control, but not in the wild type or water control. M, DNA marker; WT, wild type; H, water control; P, plasmid positive control; 1–14, transgenic foxtail millet lines.

The foxtail millet cultivar Ci846 was genetically transformed using an *Agrobacterium-mediated* callus infection method. The infected calli were screened on MS medium supplemented with hygromycin, and the transformation procedure is shown in Figure 4A. Primers specific to the hygromycin resistance gene on the vector were designed, and PCR amplification followed by sequencing was performed using genomic DNA from resistant foxtail millet seedlings as the template. As shown in Figure 4B, a 471 bp hygromycin resistance gene fragment was successfully amplified in both the positive control (plasmid) and the transgenic plants, whereas no amplification was detected in the wild type. In total, 14 hygromycin-resistant foxtail millet seedlings were obtained, indicating that the *SiMAPKKK17* overexpression construct had been successfully introduced into foxtail millet plants. These results confirmed the successful generation of transgenic foxtail millet plants carrying the *SiMAPKKK17* overexpression construct. The obtained transgenic lines were subsequently used for expression analysis and drought tolerance evaluation.

### 2.5. Expression Verification and Phenotypic Analysis of SiMAPKKK17 Overexpression Lines Under Drought Stress

**Figure 5:**
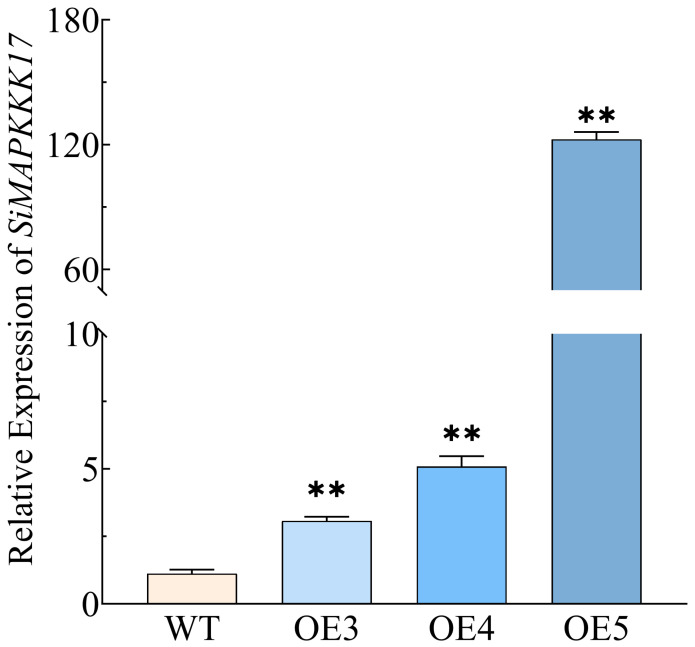
Relative expression levels of *SiMAPKKK17* in wild-type and overexpression lines. Data are presented as mean ± SE (*n* = 3 pots). Error bars indicate standard errors. Statistical significance was analyzed using IBM SPSS Statistics 26.0 by one-way ANOVA. Asterisks indicate significant differences compared with WT (** *p* < 0.01).

To assess *SiMAPKKK17* overexpression in transgenic foxtail millet, RT-PCR analysis was performed on wild-type (WT) foxtail millet cv. Ci846 and three transgenic lines (OE-3, OE-4, and OE-5). The results showed that *SiMAPKKK17* transcript levels were lowest in WT, whereas all three overexpression lines exhibited markedly higher expression, with OE-5 displaying the highest transcript abundance (Figure 5).

**Figure 6:**
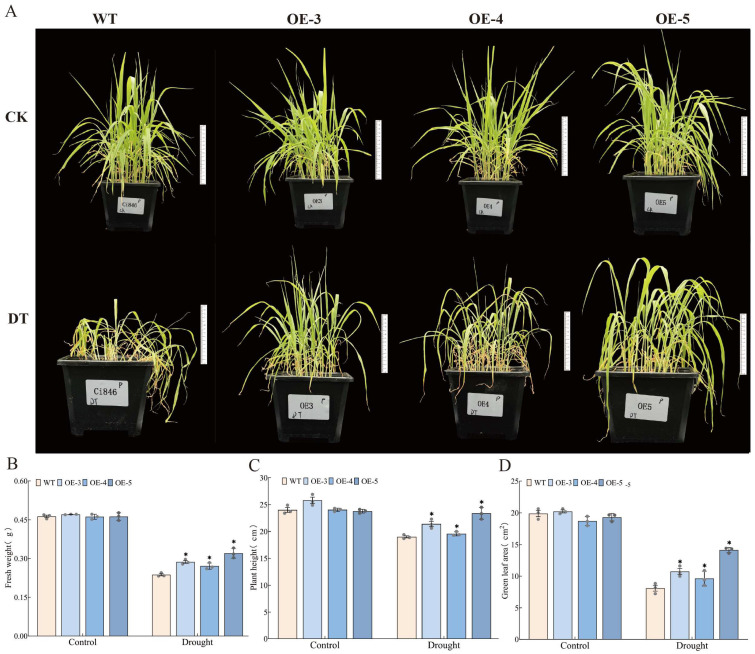
Phenotypic comparison of WT and *SiMAPKKK17* overexpression lines under normal and drought conditions. (**A**) Phenotypic performance of WT and overexpression lines after drought treatment. (**B**) Above-ground fresh weight. (**C**) Plant height. (**D**) Green leaf area. Data are presented as mean ± SE (*n* = 3 pots). Error bars indicate standard errors. Statistical significance was analyzed using IBM SPSS Statistics 26.0 by one-way ANOVA. Asterisks indicate significant differences compared with WT (* *p* < 0.05).

Based on these results, WT and the overexpression lines were subjected to drought stress. Under normal conditions, no significant differences in growth were observed between the *SiMAPKKK17* overexpression lines and wild-type (WT) foxtail millet plants. Under drought conditions, the upper leaves of WT plants showed severe wilting and necrosis, whereas the top three leaves of the overexpression lines remained green and upright (Figure 6A). Compared with WT, the above-ground fresh weight of OE-3, OE-4, and OE-5 increased by approximately 12.6%, 3.0%, and 23.0%, respectively (Figure 6B). Plant height increased by approximately 32.8%, 19.0%, and 74.5%, respectively (Figure 6C), and green leaf area increased by approximately 19.2%, 12.7%, and 33.4%, respectively (Figure 6D).

### 2.6. Transcriptome Analysis of Overexpressing Transgenic Seedling Leaves Under Drought Stress

**Figure 7:**
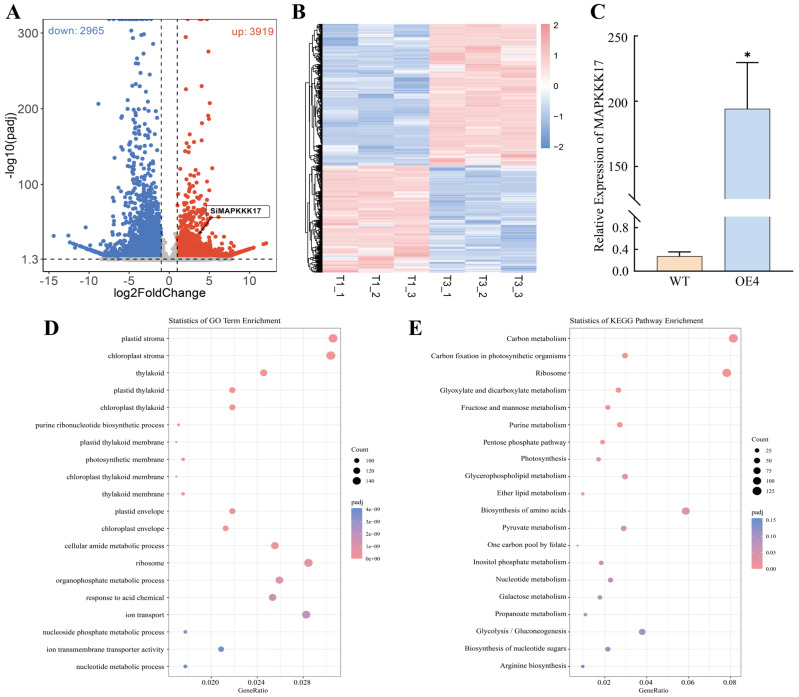
Differential Gene Expression and GO/KEGG Enrichment Analysis of *SiMAPKKK17* Transgenic Lines and Wild-type Under Drought Stress. (**A**) Volcano Plot of Differentially Expressed Genes. (**B**) Heatmap of Differentially Expressed Genes. (**C**) The expression level of the MAPKKK gene calculated by FPKM (*n* = 3, * *p* < 0.05). (**D**) GO Enrichment Plot of Differentially Expressed Genes. (**E**) KEGG Enrichment Plot of Differentially Expressed Genes.

As shown in Figure 7A, a volcano plot was used to visualize the distribution of differentially expressed genes (DEGs) between WT and OE4 under drought stress. Using |log_2_FC| ≥ 1 and an adjusted *p*-value < 0.05 as screening criteria, a total of 3919 upregulated and 2965 downregulated DEGs were identified in OE4 compared with WT. Hierarchical clustering analysis further revealed that WT and OE4 exhibited clearly distinct and opposite expression patterns, while biological replicates within each group showed high consistency (Figure 7B). Moreover, transcriptome analysis confirmed that the relative expression level of *SiMAPKKK17* was significantly higher in the OE4 transgenic line than in WT (Figure 7C), validating the successful overexpression of *SiMAPKKK17* and supporting its positive association with drought tolerance.

GO enrichment analysis indicated that the DEGs were primarily associated with cellular component terms related to chloroplast structures, including plastid stroma, chloroplast stroma, thylakoid, plastid thylakoid membrane, chloroplast thylakoid membrane, photosynthetic membrane, and chloroplast envelope. Regarding biological processes and molecular functions, the DEGs were mainly enriched in purine ribonucleotide biosynthetic processes, cellular amide metabolism, organophosphate metabolism, ion transport, nucleoside phosphate metabolism, and ion transmembrane transporter activity (Figure 7D). KEGG pathway enrichment analysis revealed that the DEGs were significantly enriched in pathways related to carbon metabolism, carbon fixation in photosynthetic organisms, ribosome, glycolate and dicarboxylate metabolism, fructose and mannose metabolism, purine metabolism, the pentose phosphate pathway, photosynthesis, glycerophospholipid metabolism, ether lipid metabolism, amino acid biosynthesis, pyruvate metabolism, one-carbon pool by folate, inositol phosphate metabolism, nucleotide metabolism, galactose metabolism, propanoate metabolism, glycolysis/gluconeogenesis, biosynthesis of nucleotide sugars, and arginine biosynthesis (Figure 7E). These results suggest that overexpression of *SiMAPKKK17* may enhance drought tolerance by regulating genes involved in photosynthesis, carbon metabolism, nucleotide metabolism, and transport-related processes.

### 2.7. GSEA Was Conducted to Profile Shifts in GO and KEGG Pathways Associated with SiMAPKKK17 Overexpression

**Figure 8:**
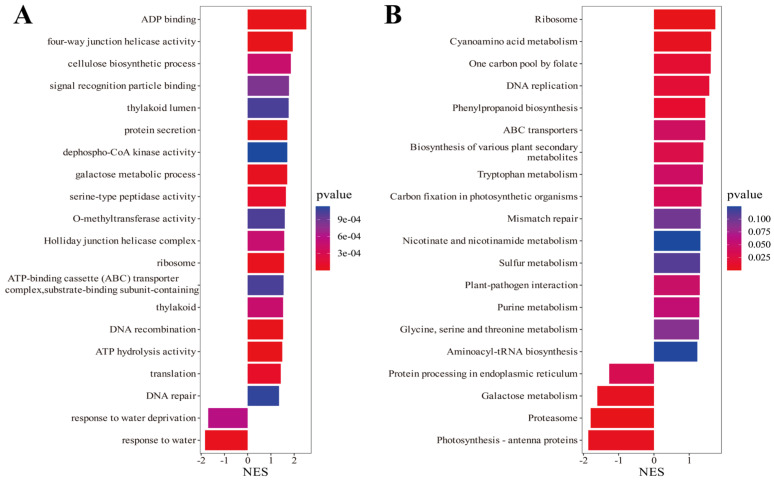
Gene Set Enrichment Analysis (GSEA) of *SiMAPKKK17*-overexpressing plants. (**A**) GSEA of GO terms. (**B**) GSEA of KEGG pathways. NES > 1, normalized enrichment score; *p* < 0.05.

To elucidate the molecular mechanisms underlying *SiMAPKKK17*-mediated drought tolerance, Gene Set Enrichment Analysis (GSEA) was performed on GO terms and KEGG pathways. GO-based GSEA (Figure 8A) revealed that *SiMAPKKK17* overexpression upregulated gene sets associated with ADP binding, cellulose biosynthetic processes, and signal recognition particle binding, indicating enhanced nucleotide binding activity and cell wall biosynthesis/reinforcement, which may confer structural resilience under water-deficit conditions. Conversely, gene sets related to response to water deprivation and DNA repair were downregulated, suggesting a modulation of canonical water-stress perception and damage-response pathways. KEGG-based GSEA (Figure 8B) showed upregulation of ribosome, one-carbon pool by folate, and DNA replication pathways, reflecting active protein synthesis, one-carbon metabolism, and cell proliferation essential for sustained growth under stress. Meanwhile, photosynthesis–antenna proteins and proteasome pathways were suppressed, implying a strategic resource reallocation from light harvesting toward stress-adaptive processes. Collectively, these results demonstrate that *SiMAPKKK17* overexpression orchestrates broad transcriptional reprogramming—coordinating cell wall integrity, metabolic activity, and stress signaling—to enhance drought tolerance.

## 3. Discussion

The MAPKKK family is a crucial upstream component of the MAPK signaling pathway, responsible for perceiving extracellular signals and activating downstream MAPKKs, thereby transmitting signals into the cell [27,28,29]. The plant MAPKKK family comprises numerous members and can be classified into three subfamilies—MEKK-like, RAF-like, and ZIK—based on kinase domain sequences and activation mechanisms. Among these, the RAF-like subfamily is commonly activated by second messengers (e.g., Ca^2+^ and ROS) and hormones such as ABA and is critically involved in drought responses. Some RAF-like members can directly phosphorylate core ABA signaling components, such as SnRK2s [30,31,32]. Several studies have demonstrated the involvement of RAF-like MAPKKKs in abiotic stress responses. Collectively, these studies indicate that RAF-like MAPKKKs often function as upstream “signal relays” that convert ABA or second-messenger cues into downstream kinase activation, providing a mechanistic context for interpreting our *SiMAPKKK17* results. For example, in wheat, TaHT1 (a RAF subfamily kinase) regulates drought tolerance through interaction with SnRK2.10 [33]. Similarly, drought- and ABA-responsive RAF-like MAPKKKs have been reported in crops such as cassava and maize, and overexpression of certain RAF-like kinases (e.g., MdRaf5) enhances drought tolerance in heterologous systems [34,35,36]. Together, these findings suggest that stress-induced expression and/or altered kinase signaling of RAF-like MAPKKKs is frequently associated with drought adaptation; however, the specific downstream outputs may differ across species and therefore require direct validation in foxtail millet.

This study provides a systematic analysis of the sequence characteristics, evolutionary relationships, and protein structure of *SiMAPKKK17* in foxtail millet. The protein consists of 486 amino acids, while its homologs range from 377 to 546 amino acids, indicating overall structural conservation with potential divergence in non-core regions. Domain analysis revealed that *SiMAPKKK17* contains a conserved STKc_MAPKKK kinase domain enriched in ATP-binding and substrate phosphorylation sites, forming the catalytic core of the protein. Phylogenetic analysis clustered *SiMAPKKK17* with MAPKKK17 homologs from Poaceae species (sorghum, maize, rice, and wheat) in Clade A, with high bootstrap support (0.773–0.935). The closest relationship was observed with sorghum SbMAPKKK17, consistent with their classification within the Panicoideae subfamily. Subcellular localization analysis demonstrated that *SiMAPKKK17* is distributed in both the plasma membrane and nucleus. This dual localization supports a role in early stress- or ABA-associated signal relay at the membrane and subsequent regulation of nuclear outputs. Considering the established roles of RAF-like MAPKKKs in ABA and abiotic stress signaling, *SiMAPKKK17* may function as a signaling bridge that transmits stress cues from the plasma membrane to the nucleus. Notably, the localization result does not by itself prove direct transcriptional control; rather, it supports a model in which *SiMAPKKK17* could modulate nuclear gene expression via phosphorylation-dependent signaling, consistent with the transcriptional reprogramming observed in our overexpression lines. Based on these findings, we speculate that *SiMAPKKK17* acts as a regulatory bridge in ABA- or drought-related pathways. While the kinase domain is conserved, variation in non-core regions may contribute to species-specific interactions and downstream outputs, underscoring the need for direct genetic and biochemical validation.

MAPK cascades (MAPKKK → MAPKK → MAPK) function as central signal amplification modules that transmit drought signals to downstream transcriptional networks, thereby inducing stress-responsive gene expression and enhancing drought tolerance [37,38,39]. Recent studies indicate that MAPKKK17 and MAPKKK18 play conserved roles in drought responses, although the degree of functional conservation varies among species. Across diverse plants, genetic and network-based evidence supports MAPKKK17/18-related modules in drought- and ABA-responsive pathways, including MAPKKK18–MAPKK3 signaling in *Arabidopsis*, MAPKKK17 as a drought-network hub in rice, AIMK1-mediated regulation in pepper, and downstream MKK contributions to ABA accumulation and drought resistance in cotton [25,26,40,41]. These findings suggest that MAPKKK17/18 may function as upstream amplification nodes in drought signaling pathways. Combined with our phylogenetic placement of *SiMAPKKK17* within Poaceae MAPKKK17 homologs, the literature supports—but does not guarantee—that *SiMAPKKK17* could operate in a similar drought-associated signaling module in foxtail millet. Accordingly, the key question addressed by our overexpression analysis is whether elevating *SiMAPKKK17* expression is sufficient to shift physiological and transcriptional drought responses toward enhanced stress tolerance.

To validate its function, *SiMAPKKK17* overexpression lines were generated in foxtail millet. Under drought conditions, WT plants exhibited severe wilting and necrosis of upper leaves, whereas OE-3, OE-4, and OE-5 maintained greener and more upright foliage. Compared with WT, the overexpression lines displayed increases of 12.86% in fresh weight, 42.08% in plant height, and 21.77% in green leaf area, indicating improved growth maintenance under stress. These phenotypes suggest that *SiMAPKKK17* promotes drought tolerance primarily by sustaining growth and leaf greenness rather than solely activating damage-response pathways. Transcriptome analysis of OE-4 versus WT identified 4580 upregulated and 3299 downregulated genes. GO enrichment revealed that many DEGs were associated with chloroplast-related structures and ion transmembrane transport activity. KEGG pathway analysis indicated significant enrichment in photosynthesis, carbon fixation, carbon metabolism, the pentose phosphate pathway, ribosome, purine metabolism, and amino acid biosynthesis. The convergence of these pathways provides a mechanistic explanation for the greener-leaf phenotype, consistent with improved biomass retention and reduced stress-induced senescence. Notably, the “Plant MAPK signaling pathway” was not significantly enriched in KEGG analysis, likely reflecting that MAPK regulation is predominantly phosphorylation-driven and may not be fully captured by DEG-based enrichment, or that only a subset of MAPK components exhibits transcriptional changes under the tested conditions. RT-qPCR further confirmed higher *SiMAPKKK17* expression in the overexpression lines, along with enhanced induction of MAPK-related and stress-responsive genes under drought. Together, the transcriptomic and RT-qPCR results support a model in which *SiMAPKKK17* modulates drought responses by reshaping downstream transcriptional programs—particularly those linked to photosynthesis, metabolism, and ion transport—while the canonical MAPK cascade may be regulated primarily at the post-translational level. In summary, *SiMAPKKK17* overexpression improves drought-associated growth maintenance in foxtail millet and induces broad transcriptional reprogramming of genes involved in photosynthesis, metabolism, and ion transport. However, the current evidence is largely based on overexpression and transcriptional outputs, so causality at the kinase-cascade level remains to be demonstrated. Additionally, some transcriptome changes may be secondary to improved growth status; thus, tracking early phosphorylation events will be essential to distinguish primary *SiMAPKKK17*-dependent signaling from downstream physiological effects. Whether *SiMAPKKK17* directly activates the canonical MAPK cascade through phosphorylation and its specific downstream targets requires further genetic and biochemical validation. Based on the phenotypic improvement and consistent transcriptomic shifts observed here, we conclude that *SiMAPKKK17* functions as a positive regulator of drought tolerance in foxtail millet, likely acting upstream to coordinate stress-responsive signaling and downstream metabolic and physiological maintenance. Future studies employing loss-of-function mutants, MAPK activation assays, and identification of direct substrates and interacting partners will be critical to resolve the precise molecular mechanisms.

## 4. Materials and Methods

### 4.1. Plant Materials

The drought-tolerant foxtail millet germplasm “JMK10” was used for cloning *SiMAPKKK17* and is maintained in our laboratory. Foxtail millet germplasm Ci846 was employed for genetic transformation and drought tolerance evaluation and was kindly provided by Dr. Xianmin Diao’s research group at the Institute of Crop Sciences, Chinese Academy of Agricultural Sciences. *Nicotiana benthamiana* was used for subcellular localization assays. Unless otherwise specified, plants used for comparative experiments were grown under identical controlled conditions and sampled at the same developmental stage.

### 4.2. Strains and Plasmids

*Escherichia coli* DH5α was used for plasmid propagation. *Agrobacterium tumefaciens* EHA105 was employed for both transient expression in *Nicotiana benthamiana* and stable transformation of foxtail millet. The overexpression vector backbone pBWA(V)HU and the subcellular localization vector pCAMBIA2300-GFP were used in this study. For *Agrobacterium* selection, LB plates were supplemented with kanamycin (50 mg/L) and rifampicin (20 mg/L) and incubated at 28 °C for 48 h in the dark.

### 4.3. Cloning of SiMAPKKK17

Based on the published sequence of *SiMAPKKK17* (*Seita.5G284400*), gene-specific primers were designed (Table 1). Total RNA was extracted from young leaves of JMK10 and reverse-transcribed into cDNA, which served as the template for PCR amplification of the *SiMAPKKK17* coding sequence. The amplified fragment was verified by Sanger sequencing and subsequently used for vector construction.

### 4.4. Bioinformatics Analyses of SiMAPKKK17

The *SiMAPKKK17* protein sequence was retrieved from *Setaria-DB*. Homologous proteins from representative Poaceae species were identified using NCBI BLASTP(2.15.0). Multiple sequence alignment was performed with MAFFT. Phylogenetic analysis was conducted using IQ-TREE 3(3.1.0) with automatic model selection, and branch support was estimated using 1000 ultrafast bootstrap replicates and 1000 SH-aLRT replicates. The resulting phylogenetic tree was visualized with iTOL(v6.x). The three-dimensional structure of *SiMAPKKK17* was predicted using SWISS-MODEL server. Conserved domains were identified using NCBI-CDD and visualized with TBtools(v2.400), while conserved residues were displayed using ESPript 3. Collinearity analysis was performed using JCVI v1.6.4.

### 4.5. In Silico Tissue Expression Analysis

Expression data for *SiMAPKKK17* across different foxtail millet tissues and developmental stages were retrieved from *Setaria-DB*. Tissue-specific expression patterns were analyzed using the available database resources.

### 4.6. Subcellular Localization of SiMAPKKK17 Protein in N. benthamiana

The coding sequence of *SiMAPKKK17* was cloned into pCAMBIA2300-GFP to generate the fusion construct pCAMBIA2300-GFP-SiMAPKKK17, which was introduced into *Agrobacterium tumefaciens* EHA105 and confirmed by PCR and sequencing.

For transient expression, *Nicotiana benthamiana* plants were grown for 4–5 weeks under a 16 h light/8 h dark photoperiod at 28 °C. *Agrobacterium* cultures harboring pCAMBIA2300-GFP-*SiMAPKKK17* or the empty pCAMBIA2300-GFP vector were resuspended in infiltration buffer and adjusted to OD600 = 1.4. Acetosyringone was added to a final concentration of 150 μM, and the suspensions were incubated for 1–1.5 h prior to infiltration. Fully expanded leaves were infiltrated from the abaxial side using a needleless syringe. Fluorescence signals were observed 36–48 h post-infiltration using a Carl Zeiss LSM 980 confocal laser scanning microscope (Carl Zeiss, Germany). GFP was excited at 488 nm and detected at 495–523 nm, whereas mCherry was excited at 561 nm. PIP2A-mCherry was used as the plasma membrane marker. For plasmolysis analysis, infiltrated leaf tissues were treated with 1 M mannitol for approximately 10 min prior to imaging.

### 4.7. Construction of the Overexpression Vector pBWA(V)HU-SiMAPKKK17

**Figure 9:**
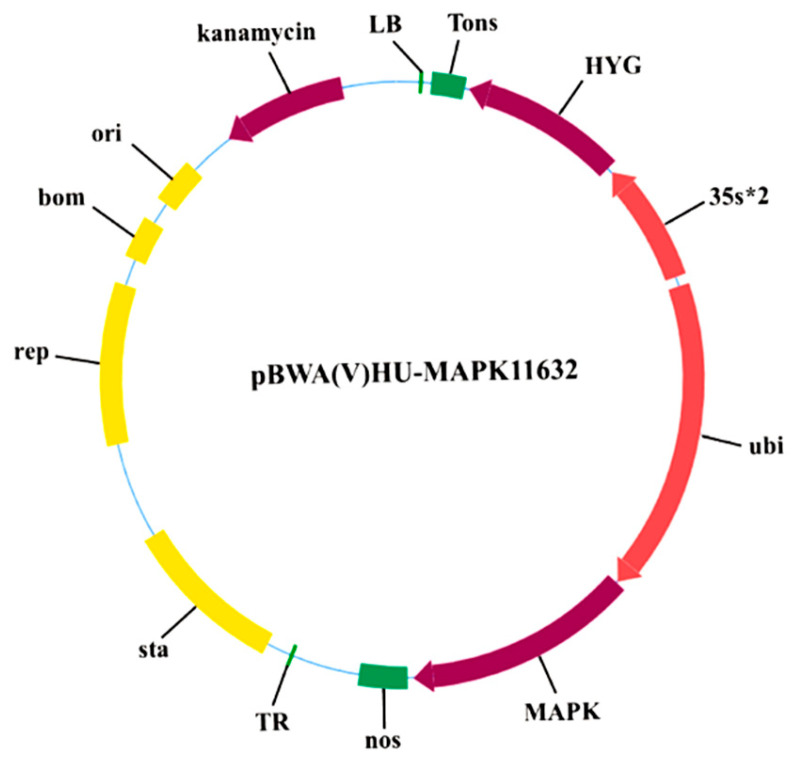
(V)HU-*SiMAPKKK17* overexpression vector map.

The *SiMAPKKK17* coding sequence was cloned into the pBWA(V)HU vector using Golden Gate cloning. Positive recombinant clones were identified by colony PCR and confirmed by sequencing. The verified construct was introduced into *Agrobacterium tumefaciens* EHA105 for foxtail millet transformation. Briefly, 1 µL of plasmid DNA was added to 50 µL of competent EHA105 cells, mixed thoroughly, and transferred to an electroporation cuvette. Following electroporation, 1 mL of LB medium was added, mixed, and transferred to a 1.5 mL microcentrifuge tube for recovery at 30 °C with shaking at 180 rpm for 30 min. Subsequently, 50 µL of the recovered culture was spread evenly onto LB solid medium containing 50 mg/L kanamycin and 20 mg/L rifampicin and incubated in the dark at 28 °C for 48 h to select positive *Agrobacterium* colonies (Figure 9). The resulting transformants were used for *Agrobacterium-mediated* foxtail millet transformation.

### 4.8. Transformation of Agrobacterium and Generation of Transgenic Foxtail Millet

The verified pBWA(V)HU-*SiMAPKKK17* construct was introduced into *Agrobacterium tumefaciens* EHA105 and used for foxtail millet transformation. *Agrobacterium-mediated* transformation of foxtail millet (Ci846 background) was performed by Wuhan Boyuan Biotechnology Co., Ltd. (Wuhan, China) using an established protocol. Embryogenic calli derived from Ci846 were used as explants for transformation. The calli were infected with *A. tumefaciens* harboring the overexpression construct at an OD_600_ of 0.1 for 10 min. Both the infection and co-cultivation media were supplemented with 0.02% (*w*/*v*) acetosyringone, and explants were co-cultivated for 3 d at 22 °C in the dark. Transformed tissues were selected using hygromycin (30 mg/L), and bacterial overgrowth was suppressed with cefotaxime (200 mg/L). Regeneration and rooting were carried out on appropriate media for approximately 20 d, after which regenerated plantlets were transferred to soil and grown to maturity.

In this study, T_0_ plants refer to the primary regenerated transformants, whereas T_1_ plants represent the progeny grown from self-pollinated seeds of T_0_ plants. The zygosity of T_1_ plants was not determined.

### 4.9. Molecular Identification of Transgenic Plants

Genomic DNA was extracted from foxtail millet leaves using the CTAB method. Transgenic plants were identified by PCR amplification of the hygromycin resistance gene (*hpt*) using the primers F: 5′-AAATCCGCGTGCACGAGGT-3′ and R: 5′-TCGTTATGTTTATCGGCACTTTGCA-3′. The pBWA(V)HU-*SiMAPKKK17* plasmid served as the positive control, while WT Ci846 and ddH_2_O were used as negative controls.

### 4.10. Drought Tolerance Evaluation of Transgenic Foxtail Millet Plants

Drought stress experiments were conducted using WT Ci846 and three *SiMAPKKK17* overexpression lines (OE-3, OE-4, and OE-5). Seeds were surface-sterilized with 0.5% NaClO, rinsed three times with sterile water, and sown in 25-cm-diameter pots containing a 1:1 (*v*/*v*) mixture of nutrient soil and vermiculite. Plants were grown in a controlled chamber under a 10 h light/14 h dark photoperiod at 30 °C/26 °C. Drought treatment was initiated at the six-leaf stage. For each genotype, well-watered control and drought treatment groups were established. Control plants were maintained at 70% ± 10% soil moisture, whereas drought-treated plants received no additional watering after the final full irrigation. Soil relative moisture content was monitored daily by weighing. Leaf samples were collected 5 days after drought initiation, immediately frozen in liquid nitrogen, and stored at −80 °C.

#### 4.10.1. Phenotypic Changes

Above-ground fresh weight, plant height, and green leaf area were measured with three biological replicates per treatment. Plant height was determined using a ruler, and above-ground fresh weight was recorded after removal of roots. Green leaf area was estimated using the formula leaf length × leaf width × 0.75, with leaves retaining more than half of their area as green counted as green leaves.

#### 4.10.2. Transcriptomic Measurement and Data Analysis

Leaf samples from WT and the *SiMAPKKK17* overexpression line OE-4 under drought treatment were subjected to RNA sequencing. RNA quality was assessed using Qubit 4.0 (Thermo Fisher Scientific, Waltham, MA, USA), NanoDrop (Thermo Fisher Scientific, Waltham, MA, USA), and an Agilent 2100 Bioanalyzer (Agilent Technologies, Santa Clara, CA, USA). Sequencing libraries were generated and sequenced on the DNBSEQ-T7 platform (MGI Tech Co., Ltd., Shenzhen, China). Raw reads were filtered with fastp(v0.21.0) and aligned to the foxtail millet reference genome using HISAT2(v2.1.0), and read counts were obtained using featureCounts(v2.0.8). Differential expression analysis was performed with DESeq2(v1.30.1), with differentially expressed genes (DEGs) defined as padj < 0.05 and |log_2_FC| ≥ 1. Gene Ontology (GO) and Kyoto Encyclopedia of Genes and Genomes (KEGG) enrichment analyses were conducted using clusterProfiler(v3.8.1).

#### 4.10.3. qRT-PCR

Quantitative real-time PCR (qRT-PCR) was performed to validate the expression of *SiMAPKKK17*. Total RNA extraction and reverse transcription were conducted as described above. Gene-specific primers are listed in Table S1. qPCR was carried out using AceQ qPCR SYBR Green Master Mix (Vazyme Biotech Co., Ltd., Nanjing, China) on a CFX96 Real-Time PCR Detection System (Bio-Rad Laboratories, Hercules, CA, USA). Relative expression levels were normalized to the foxtail millet Actin gene (*Seita.9G269800*) and calculated using the 2^−ΔΔCt^ method. Three biological replicates were analyzed, each with three technical replicates.

### 4.11. Statistical Analysis

Data are presented as mean ± standard deviation (SD). Statistical analyses were performed using GraphPad Prism 10.1.2. Comparisons between two groups were conducted using Student’s *t*-test, while multiple-group comparisons were analyzed by one-way ANOVA followed by Tukey’s multiple-comparison test. Differences were considered statistically significant at *p* < 0.05.

## 5. Conclusions

In this study, *SiMAPKKK17* was identified as a positive regulator of drought tolerance in foxtail millet. Subcellular localization analysis revealed that the protein is distributed in both the plasma membrane and the nucleus. Functional characterization demonstrated that overexpression of *SiMAPKKK17* significantly enhanced drought tolerance, with transgenic lines exhibiting improved growth maintenance under drought stress. Transcriptome and qRT-PCR analyses further indicated that *SiMAPKKK17* overexpression is associated with altered expression of genes involved in ion transport, chloroplast function, and metabolic homeostasis. Collectively, these findings provide new evidence for the role of *SiMAPKKK17* in drought adaptation in foxtail millet. Nevertheless, the precise downstream mechanisms through which *SiMAPKKK17* regulates drought responses remain to be elucidated.

## Figures and Tables

**Table 1:** Primer Sequences Used in This Study.

Primer Name	Sequence	Application
*SiMAPKKK17*-F	CAGTGGTCTCACAACATGGTGATGATGAAGCAGCTCCGG	Gene cloning
*SiMAPKKK17*-R	CAGTGGTCTCATACACTACCTTGCTAACGATGGTACGGC	
pBWA(V)HU-*SiMAPKKK17*-F	CCTGCCTTCATACGCTATTTATTTGCTTGG	Vector construction
pBWA(V)HU-*SiMAPKKK17*-R	GTACCCGATCCGGTGGACC	
*Seita.5G284400*-F	GCCATTAGCCAGGCCAGTTA	RT-qPCR
*Seita.5G284400*-R	GTTCCTGTTACAAGCACCGC	
*Actin-*F	AAGGAGATCACTGCCCTTGC	
*Actin*-R	TCCTGTGGACAATTGCTGGG	

## Data Availability

The original contributions presented in this study are included in the article. Further inquiries can be directed to the corresponding authors.

## Supplementary Materials

The following supporting information can be downloaded at https://www.mdpi.com/article/10.3390/plants15071055/s1.
