# Supplementary material for: Cloning and Functional Analysis of the SiMAPKKK17 Gene in Foxtail Millet (Setaria italica)
Source: Plants (Basel). 2026 Mar 30;15(7):1055. doi: 10.3390/plants15071055 (PMC13074924; doi:10.3390/plants15071055)
Supplement: Supplementary file 1 [file plants-15-01055-s001.zip › plants-4161895-supplementary.pdf]

Table S1 List of RT-qPCR primers

Gene id	Gene	Forward sequence (5'~3')	Reverse sequence (5'~3')	PCR Products(bp)
5G284400	*MAPKKK17*	GCCATTAGCCAGGCCAGTTA	GTTCCTGTTACAAGCACCGC	120
Actin	*Actin*	AAGGAGATCACTGCCCTTGC	TCCTGTGGACAATTGCTGGG	175
